# Stimulation of α_1a_ Adrenergic Receptors Induces Cellular Proliferation or Antiproliferative Hypertrophy Dependent Solely on Agonist Concentration

**DOI:** 10.1371/journal.pone.0072430

**Published:** 2013-08-22

**Authors:** Beilei Lei, Debra A. Schwinn, Daniel P. Morris

**Affiliations:** 1 Department of Anesthesiology, Duke University Medical Center, Durham, North Carolina, United States of America; 2 Departments of Anesthesiology, Pharmacology, Biochemistry, University of Iowa Carver College of Medicine, Iowa City, Iowa, United States of America; 3 Department of Anesthesiology and Pain Medicine, University of Washington, Seattle, Washington, United States of America; University of São Paulo, Brazil

## Abstract

Stimulation of α_1a_Adrenergic Receptors (ARs) is known to have anti-proliferative and hypertrophic effects; however, some studies also suggests this receptor can increase cell proliferation. Surprisingly, we find the α_1a_AR expressed in rat-1 fibroblasts can produce either phenotype, depending exclusively on agonist concentration. Stimulation of the α_1a_AR by high dose phenylephrine (>10^−7^ M) induces an antiproliferative, hypertrophic response accompanied by robust and extended p38 activation. Inhibition of p38 with SB203580 prevented the antiproliferative response, while inhibition of Erk or Jnk had no effect. In stark contrast, stimulation of the α_1a_AR with low dose phenylephrine (∼10^−8^ M) induced an Erk-dependent increase in cellular proliferation. Agonist-induced Erk phosphorylation was preceded by rapid FGFR and EGFR transactivation; however, only EGFR inhibition blocked Erk activation and proliferation. The general matrix metalloprotease inhibitor, GM6001, blocked agonist induced Erk activation within seconds, strongly suggesting EGFR activation involved extracellular triple membrane pass signaling. Erk activation required little Ca^2+^ release and was blocked by PLCβ or PKC inhibition but not by intracellular Ca^2+^ chelation, suggesting Ca^2+^ independent activation of novel PKC isoforms. In contrast, Ca^2+^ release was essential for PI3K/Akt activation, which was acutely maximal at non-proliferative doses of agonist. Remarkably, our data suggests EGFR transactivation leading to Erk induced proliferation has the lowest activation threshold of any α_1a_AR response. The ability of α_1a_ARs to induce proliferation are discussed in light of evidence suggesting antagonistic growth responses reflect native α_1a_AR function.

## Introduction

Adrenergic Receptor (AR) stimulation by epinephrine has been recognized as integral to the fight or flight response [1] of the sympathetic nervous system since early in the 20^th^ century [2]. As part of the sympathetic response, these receptors are activated within seconds of stimulus recognition; however, they are also involved in more extended processes including tissue injury and repair. Early studies distinguished the α_1_AR family from the α_2_ARs and βARs on a pharmacological basis using specific agonists and inhibitors [3]. Even before identification of the DNA sequences encoding the three α_1_AR genes, it was recognized that α_1_ARs induced smooth muscle contraction through Ca^2+^ release [4] directed by phospholipase C beta (PLCβ).

Canonical α_1a_AR signaling is initiated by agonist stimulation that allows GTP association with Gq, dissociation of the trimeric G proteins and activation of PLCβ via direct interaction with Gq/GTP [5]. Resultant cleavage of membrane-bound phosphatidylinositol 4,5 bisphosphate (PIP2) produces soluble inositol triphosphate (IP3) and membrane-bound diacyl glycerol (DAG). In most cells, IP3 induces acute release of intracellular Ca^2+^ stores through opening of the IP3R channel, while membrane bound DAG activates novel protein kinase C (PKC) isoforms (δ, ε, η, μ and θ) and in combination with Ca^2+^; activates four typical PKC isoforms (α, β_I_, β_II_, γ). DAG can also induce Ca^2+^ entry from the extracellular medium through canonical transient receptor potential channels [6], while depletion of ER Ca^2+^ stores can lead to store operated Ca^2+^ entry through calcium release activated calcium channels [7]. Regulation of these [8], and probably other [9] channels, produce the extended increase in cytosolic Ca^2+^ associated with α_1a_AR activation [10]–[13]. In addition, Gq appears to directly activate signaling through effectors including GRK2 [14] and RhoGEFs [15] with the later activating Rho/Raf GTPases. Although limited information is available for the α_1_ARs, stimulation of GPCRs also activates Gβγ subunits, which signal through a variety of molecules including some isoforms of PLCβ [16]. In addition, Gq-coupled receptors can transactivate EGFR and other Receptor Tyrosine Kinases through triple membrane pass (TMP) signaling that involves matrix metalloproteases cleavage of growth factor precursors [17]–[19]. Other signaling proteins reportedly activated by α_1_ARs include PKD1 [20], PLA2 [21], PLD [22], AMPK [23] and Na^+^/H^+^ exchangers [24]. Despite the extensive study, mechanisms of α_1_AR function appear to be very complex and are poorly understood in most tissues [25].

Functionally, the α_1_ARs are present in many cell types where they play diverse roles: however, attention has focused on stress responses associated with the cardiovasculature. Although α_1_AR signaling can be identified by phenylephrine (PE) activation, the subtype that produces a specific biological response can be difficult to establish in native tissues. Pharmacologic identification of the α_1a_AR is more dependable, as selective agonists and inhibitors are available for this subtype [26]. Nevertheless, transgenic mice missing individual α_1_AR subtypes have proven invaluable, although murine phenotypes can be altered by small amounts of the remaining subtypes [27], [28], as well as compensatory upregulation [29] and synergistic interactions. Almost unstudied are differences in α_1_AR subtype expression within distinct [30], [31] and similar [32] cell types of a single tissue, despite the potential importance of endocrine like growth factor release produced by transactivation.

Most studies of α_1a_AR mediated cell signaling have been performed in expression models using epitope tagged receptors not only because of the clarity provided by expression of a single subtype, but also because native receptor levels are too low for antibody detection [33]. In these models, comparison of signaling efficacy between individual subtypes has shown α_1a_AR signaling to be more robust in HeLa [10], rat-1 fibroblast [22], [34], [35], HEK293 [34], SK-N-MC (1996theroux) and CHO [36] cells, although the relationship between canonical signaling intensity and α_1_AR-induced phenotypic responses [36]–[39] remains unclear. Beyond signaling intensity, there are subtype specific mechanisms such as the rapid internalization [40] and proliferative phenotypes [38] of the α_1b_AR that contrast with the slow internalization [41] and antiproliferative [38] phenotypes α_1a_AR. In some native cells, the α_1a_AR subtype displays unique signaling complexity, apparent as pharmacologically distinct basal conformations, either with high prazosin affinity (α_1a_AR) or with low prazosin affinity (α_1a(L)_AR), sometimes observed in activity assays [26]. The low affinity phenotype, often designated as the α_1a(L)_AR, appears to be important in some prototypic α_1a_AR models of smooth muscle cell (SMC) contraction [42]–[45].

The significance of fibroblasts to injury responses involving the α_1a_AR [46], as well as chronic stress including those that produce cardiomyopathy [47], [48] has increased the need to understand α_1a_AR biology in fibroblasts. Recently, we identified a naturally occurring α_1_AR SNP from a hypertensive patient, which increases proliferation of rat-1 fibroblasts [49] through a mechanism involving constitutive transactivation of EGFR [50]. In the current study, we investigated the connections between α_1a_AR signaling pathways and biological phenotype following the discovery that wild-type α_1a_ARs can induce either anti-proliferative or proliferative responses dependent solely on differences in signaling intensity due to agonist concentration. Because clonal rat-1 fibroblasts in the same media are identical prior to low or high dose α_1a_AR stimulation, causal and coincident signaling events can be distinguished completely free of superfluous differences due to environment, clonal variation and cell type.

## Methods

### Materials

Reagents and suppliers were: phenylephrine, prazosin, U73122, PMA, GF109203X, genistein, thapsigargin, HRP-conjugated goat antimouse IgG (A9169) and antirabbit IgG (A9044), [Sigma, St. Louis, MO]; G418, calphostin C, BAPTA/AM, W-7, KN-93, PD 98059, SB 203580 (Calbiochem, San Diego, CA); ^125^I-(2-β-(4-hydroxyphenyl)-ethylamineomethyl)-tetralone ([^125^I]HEAT), [Perkin Elmer Life Sciences, Boston, MA]; High Glucose Dulbecco’s Modified Eagle Medium (11995) and Pen/Strep (15140) [Gibco, Grand Island, NY]; Hyclone Fetal Bovine Serum (SH30071.03HI), Restore stripping buffer (21059) and Supersignal substrate (34076) [Thermo Scientific, Rockford, IL].

### Cell Culture

**Figure 1:**
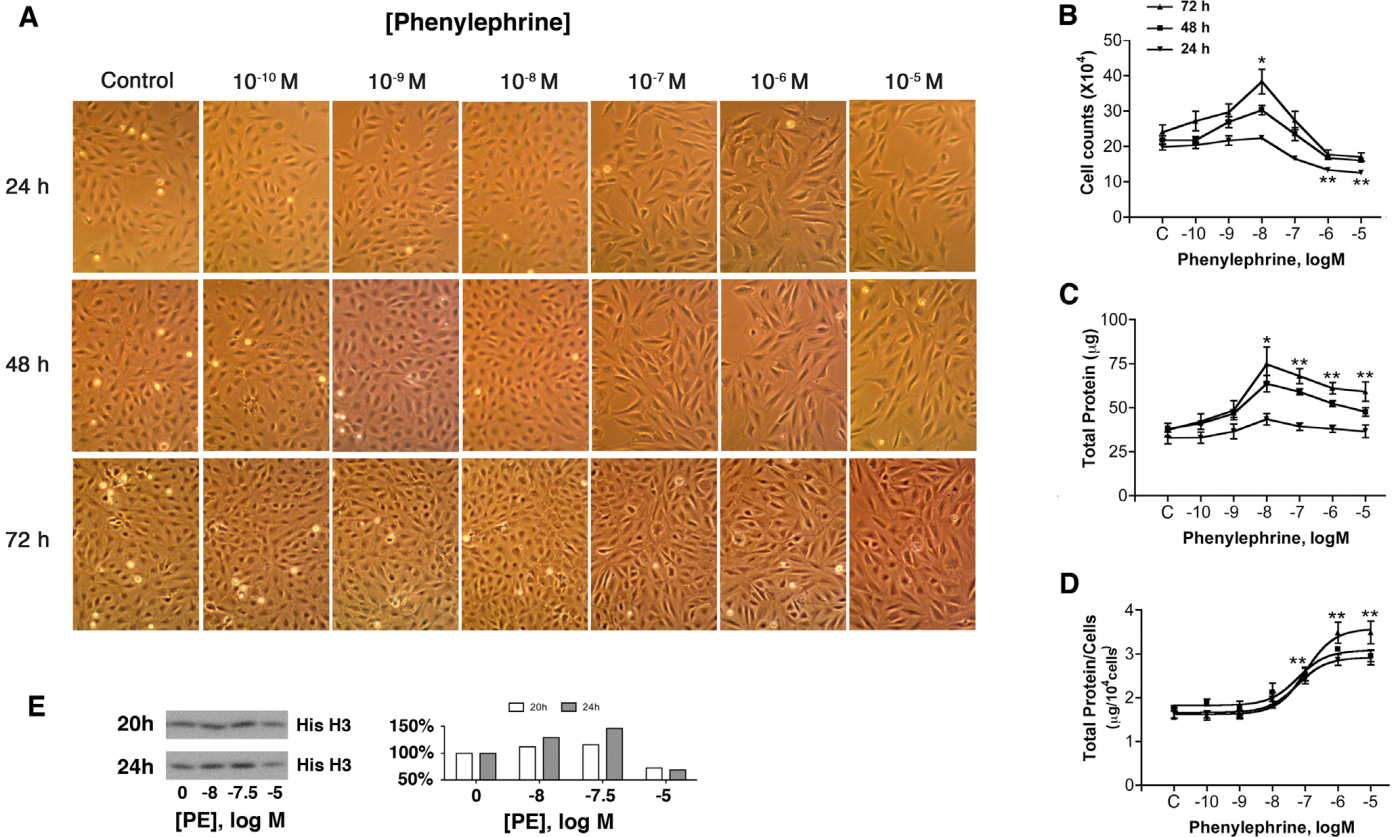
The α_1a_AR induces proliferation at low PE concentrations and antiproliferative hypertrophy at high PE concentrations. Rat-1 cells stably expressing the α_1a_AR at 1.77 pmol/mg of total protein were incubated at 37°C under 5% CO2 in serum-free DMEM for 24, 48 and 72 h with the indicated concentrations of PE (10^−10^ to 10^−5^ M). The effect of PE on α_1a_AR-induced proliferative and hypertrophic growth responses was determined by side-by-side (**A**) morphological observation, (**B**) cell counts; (*), P<0.05 at 48 and 72h; (**), P<0.05 at 24h, (**C**) protein quantitation per well; (*), P<0.05 at 48h; (**) P<0.05 at 48 and 72h, and (**D**) estimation of protein per cell; (*), P<0.05 at all times. All compared by 1-way ANOVA to basal (n = 3–8). **E** Representative Western blot images and semi-quantitative analysis of histone H3 protein levels at 20 and 24 hours.

Rat-1 cells stably expressing human, hemagglutinin (HA) tagged α_1A_-AR at about 1.77 pmol/mg of total protein [49] were maintained in complete media containing DMEM, 10% FBS, penicillin/streptomycin (P/S) and 400 µg/ml G418. Prior to all experiments, cultures near confluence but not quiescent were trypsinized and plated in 6 or 12 well plates and grown in complete media without G418 selection. For growth assays with varied PE concentrations (i.e Fig. 1) cells were washed twice with serum free (SF) media (DMEM with P/S) and then returned to SF media and immediately stimulated with the α_1_-AR selective agonist, PE, at the indicated concentrations for 1, 2 or 3 days. For Western analysis or growth assays involving pretreatment with agents, cells were washed twice with SF media and then incubated in SF media for 3–4 hours prior to pretreatment with agents and stimulation with PE for 1 day. Unless indicated, agents were added to cells 30 min Prior to PE. For Western analysis cells were plated at appropriate density in 6 well plates (100–200 thousand cells per well), grown to near confluence (80–100%), washed twice with DMEM and then incubated in DMEM for 3–4 hours prior to treatments and PE stimulation as indicated.

### Analysis of Cell Growth

For growth assays, analysis of cell morphology (imaging), cell number and protein per well were performed side-by-side. After PE stimulation, images showing typical density were captured using a digital camera. Cells were counted using a hemocytometer following a PBS wash, trypsinzation and addition of about 1 ml of DMEM. Total protein per well was always quantitated in parallel wells using the BCA protein assay reagent kit (Pierce) with BSA as a standard. Analysis was performed on 50 µl samples from PBS washed cells harvested in 250 µl of lysis buffer (1% nonidet P-40 and 0.5% sodium deoxycholate).

### Western Blotting

Cells in DMEM for 3 to 4 hours were pretreated with agents and stimulated with PE at the times and concentrations indicated prior to media aspiration and direct solubilization with 2% SDS sample buffer. Samples were sometimes frozen in Liquid N_2_, heated at 95°C for 5 min, and then subject to 15 sec of sonication or hard vortexing to shear DNA. Samples, loaded equally and resolved on 26 well 4–20% or 10–20% precast Criterion SDS gels were transferred to polyvinylidene difluoride (162–0177) or nitrocellulose (162–0015) membranes in standard buffers with 10% methanol and 0.005% SDS using a Criterion Blotter (plate), all from Bio-Rad, (Hercules, CA). Western treatment conditions used TBS with 0.1% Tween, 5–10% dry milk (Biorad) with antibodies, four 5 min, 40 ml washes, and 1h, 25°C, 2° antibody incubations. Primary antibodies (target named) were incubated at 25°C (1–3 h) or 4°C (overnight) at ≥ 1/1000 dilutions unless indicated including Erk (9102), P-Erk1/2 (9101), p38 (9212), P-p38 (9211), JNK (9255), P-JNK (9251) at 1/500, Akt (9272), P-Akt-T308 (2965), P-Atf2 (9221) and P-Mk2 (3041) from Cell Signaling Technology, (Beverly, MA) and P-FGFR3-Y724 (33041) at 1/500 from SCBT, (Santa Clara CA) or P-EGFR1068 (324867) from EMD Millipore. As required for the substrate, diluted HRP secondary antibodies were used at ∼1/50,000 with PVDF and ∼1/10,000 with nitrocellulose membranes. When possible multiply probed blots were mildly stripped with Restore stripping buffer for 5 minutes at 25°C or harshly stripped with 2% SDS plus 0.7% β-mercaptoethanol at 50°C for 30 minutes. Signal was detected with X-ray film or when noted imaged and quantitated with a HD2 CCD camera (Alpha Innotech, San Leandro, CA). Semi-quantitative analysis of band intensity from x-ray film was done with ImageJ using non-saturated exposures with membrane background.

### Statistical Analysis

Results are expressed as the mean±SEM, compiled from *n* replicate experiments each performed in duplicate or triplicate. Statistical significance was analyzed by one-way or two-way ANOVA and where identified, respective, Dunnett or Bonferri post-tests. All calculations were performed using GraphPad Prism (GraphPad Software, San Diego, CA) with *p*<0.05 considered significant.

## Results

### Biological Effects of α_1a_AR Stimulation

Although the α_1_ARs have been shown to activate a wide array of stress and growth related pathways, increased proliferation is not a commonly observed phenotype. Thus it was initially surprising when α_1a_AR stimulation by low doses of agonist increased proliferation of rat-1 fibroblasts; a frequently used model without native adrenergic receptors derived from embryonic fibroblasts [51]. In these experiments, rat-1 cells stably expressing HA-α_1a_AR were incubated for 24, 48 and 72 hours with various concentrations of PE (10^−10^ to 10^−5^ M) and the effects of agonist stimulation on proliferative and hypertrophic growth responses determined by side-by-side cell counts, total cellular protein measurement and morphological observation. As previously reported [38], [39], high doses of PE (10^−6^ to 10^−5^ M) are strongly antiproliferative and induced a visually evident decrease in cell number (Fig. 1A) quantitatively established by cell counting (Fig. 1B). This strong cell cycle blockade was accompanied by an obvious hypertrophic response [38], reflected in the near doubling of the protein per cell (Fig. 1A and 1D). In this clonal line [52], the dose dependence of this hypertrophic phenotype on PE (Fig. 1D) was similar to the dose dependence of IP3 formation (EC_50_ ∼3×10^−7^ M), which serves as a measure of G protein activation. More unexpectedly, we found low concentrations of PE (∼10^−8^ M) to be associated with increased proliferation relative to untreated control cells (Fig. 1B). Agonist dose response curves also showed higher levels of histone H3 in cells exposed to minimal PE, indicative of increased DNA synthesis [53]. This unexpected growth response prompted us to reconsider the ability of α_1a_AR to regulate p38, JNK, and Erk1/2, as these MAPKs are key regulators of cell growth.

### Activation of Stress Activated Protein Kinases

**Figure 2:**
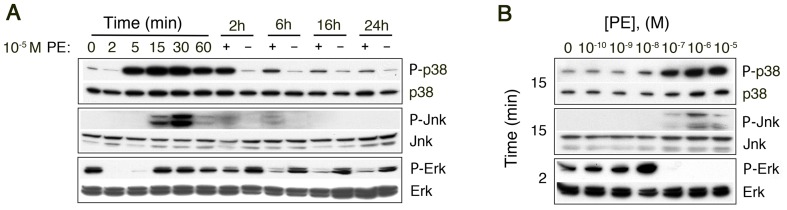
Western analysis of high dose α_1a_AR induced MAPK activation using phospho-specific antibodies for activated kinases. α_1a_AR expressing cells were placed into SF media for 3–4 hours, treated with PE for the indicated time and collected by direct addition of SDS sample buffer. (**A**) Time course following treatment with 10^−5^ M PE shows strong p38 activation, transient Jnk activation and acute inhibition of already low basal ERK activity followed by transient partial recovery. (**B**) Dose response of PE and MAPK phosphorylation. Following transfer to serum free media the cells were treated for 15 or 2 min with 0 to 10^−5^ M PE as indicated.

The stress activated MAPKs, p38 and Jnk, were both activated following stimulation of HA-α_1a_AR expressing rat-1 cells with 10^−5^ M PE (Fig. 2A, top panel). Following an acute period (2 min) where low basal p38 phosphorylation decreases modestly, p38 phosphorylation became intense within 5 minutes and maximal near 15 to 30 minutes. Thereafter p38 phosphorylation levels decreased from 1 to 24 hours, but remain above basal levels for at least 24 hours. Dose response experiments at several PE concentrations show the extent of p38 phosphorylation at 15 minutes (Fig. 2B, top panel) is also similar to the dose dependence of IP3 formation [49], [52]. Compared to p38 activation, JNK phosphorylation is both delayed [54] and more transient, reaching a maximum near 30 minutes before waning rapidly (Fig. 2A, middle panel). The PE dose dependence of Jnk phosphorylation (Fig. 2B, middle panel) displayed a profile grossly similar to p38 activation.

### High doses of PE Produce Sustained Erk Inhibition

Although α_1a_AR stimulation can produce modest Erk activation [36], in rat-1 cells high doses of PE (10^−5^ M) inhibit Erk activity (Fig. 2A, lower panel) in agreement with prior evidence [37]. Although Erk phosphorylation was severely reduced both acutely (2 min) and later in the time course (∼1 hour), we did observe a period of recovery between 15 and 30 minutes (Fig. 2A, lower panel) where Erk phosphorylation approached basal levels. At even longer times, Erk phosphorylation remained depressed relative to untreated control cells. Problematically, the basal Erk phosphorylation level of cells placed into SF media for 3–4 hours was both low and sensitive to minor details of cell handling, nevertheless, the response pattern including both minima and the recovery period was qualitatively consistent (n>13). A dose response curve directed at the time of acute Erk dephosphorylation (∼2 min), defined a broad range of agonist concentrations (10^−7^ to 10^−5^ M PE) that produced this acute inhibitory effect (Fig. 2B lower panel). In addition, this curve showed an increase in Erk phosphorylation at 10^−8^ M PE relative to basal levels, suggesting a narrow range of concentrations within which α_1a_-AR stimulation can increase Erk activity. Given the established role of Erk in enabling proliferation, the elevated phosphorylation associated with low dose agonist stimulation suggested a basis for increased proliferation (Fig. 1B) that is addressed below.

### Activation of p38 is Required for the PE-induced Proliferative Blockade

**Figure 3:**
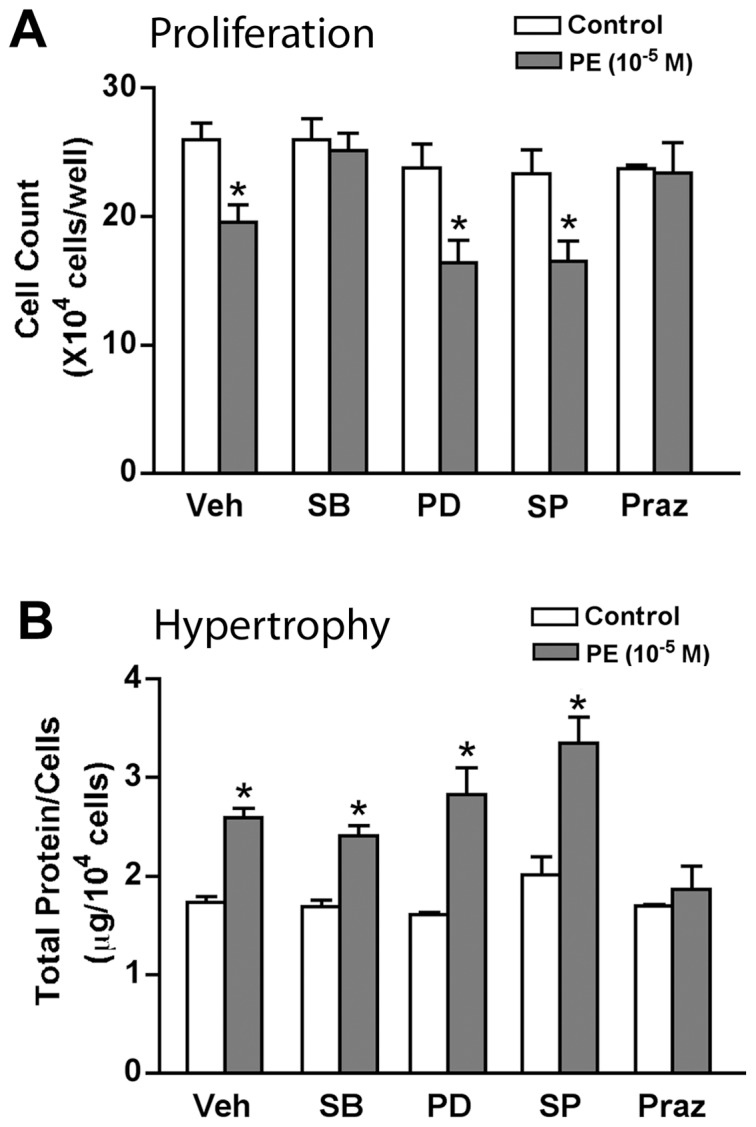
The antiproliferative effects of high dose PE require p38 activity. α_1a_AR expressing cells placed into SF media were pretreated for 30 min with vehicle (0.1% DMSO), 20 µM PD98059 (MEK/Erk inhibitor), 10 µM SB203580 (p38 inhibitor), 10 µM SP600125 (Jnk inhibitor), or 10 µM Prazosin (α_1_AR) and then stimulated with 10 µM PE for 24 h prior to (**A**) cell counting or (**B**) protein assay and estimation of cellular protein content. Values are the mean±SEM (n≥3); (*), P<0.05, compared to vehicle or inhibitor alone.

To investigate the mechanism of α_1a_AR induced, antiproliferative hypertrophy, we employed inhibitors of MAPKs, including the p38 kinase inhibitor, SB203580, the JNK inhibitor, SP600125, and the MEK inhibitor, PD98059, which blocks Erk phosphorylation. Cells in SF media were pretreated for 30 min with vehicle or inhibitors and then incubated with 10 µM PE for 24 hours. As shown above, PE at higher doses significantly inhibited cell growth (Fig. 3A) and increased cell size (Fig. 3B) relative to unstimulated control cells. As for Cho cells [36], the p38 inhibitor, SB203580, prevented the antiproliferative response induced by α_1a_-AR stimulation in rat-1 fibroblasts (Fig. 3A); however, the inhibitor had no significant effect on cell hypertrophy (Fig. 3B). Neither Erk or Jnk inhibition interfered with either PE induced phenotype; however, the α_1_AR inhibitor, prazosin, completely reverses both cell cycle blockade and hypertrophy.

### Proliferation Induced by Low doses of PE Required Erk Activation

**Figure 4:**
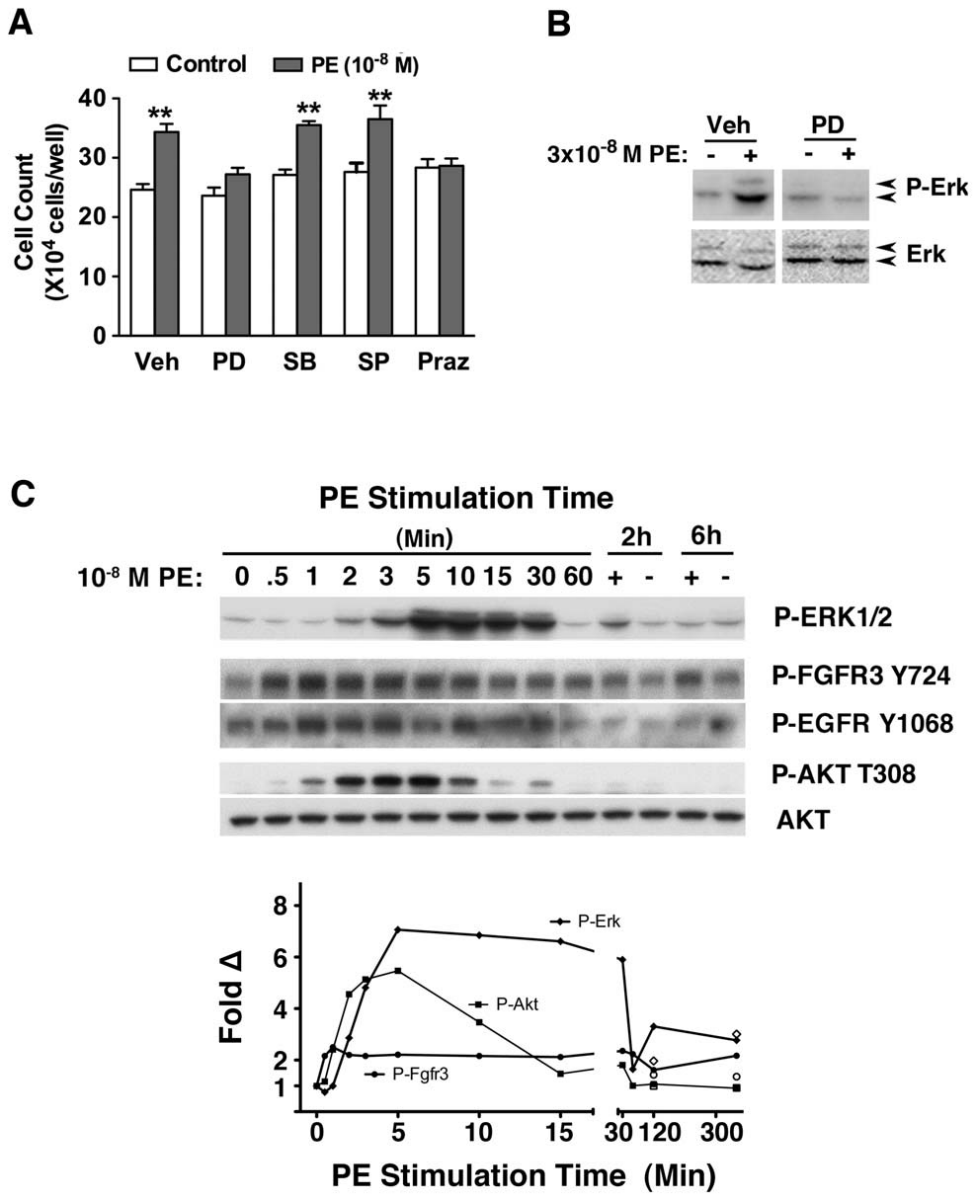
Low dose α_1a_AR stimulation activates growth-associated signaling. α_1a_AR expressing cells were placed into SF media prior to pretreatment and stimulation. (**A**) Prior to counting, cells were pretreated for 30 min with vehicle (0.1% DMSO), 20 µM PD98059 (MEK/Erk inhibitor), 10 µM SB203580 (p38 inhibitor), 10 µM SP600125 (Jnk inhibitor), or 10 µM Prazosin (α_1_AR) and then stimulated with 10 µM PE for 24 h. Values are the mean±SEM (n≥3); (*), P<0.05, compared to vehicle or inhibitor alone. (**B**) 20 µM PD98059 blocks PE-induced Erk phosphorylation. (**C**) Erk is acutely activated within 2 min of 10 µM PE addition and remains robustly phosphorylated for 15–30 minutes. Prior to Erk activation, stimulation induces sustained phosphorylation of EGFR and FGFR and acute transient phosphorylation of Akt at T308 downstream of PI3K/Akt signaling. Total Akt provides the load control for this multiply reprobed blot. In the lower panel, differences in temporal activation patterns are shown following semi-quantitative analysis of Erk, FGFR and Akt phosphorylation. Results represent the fold increase in phosphorylation relative to basal signal (Erk, FGFR3) or for Akt, background (arbitrary) signal.

Given the established importance of MAPKs in control of cell growth [55], the role of MAPKs and upstream activators in low dose α_1a_-AR signaling was investigated under conditions otherwise identical to those used in the high dose experiments. Inhibitor analysis showed that p38 and Jnk signaling were not involved; however, inhibition of Erk signaling largely blocked proliferation induced by 10^−8^ M PE (Fig. 4A) at concentrations of the MEK inhibitor, PD98059, that also prevented increased Erk phosphorylation (Fig. 4B). Consistent with control of proliferation, the addition of 10^−8^ M PE increased ERK phosphorylation within 2 minutes and produced strong activation between 5 and 30 minutes (Fig. 4C). In contrast, P-Jnk was undetectable under basal and stimulated conditions (data not shown). Stimulation with 10^−8^ M PE produced p38 phosphorylation that was marginally (e.g. Fig. 2B) but not significantly higher than detectable basal levels (1.29±0.15-fold, n = 6) and never exceeded 1.8-fold over basal in any low dose experiment.

### EGFR Transactivation Precedes and is Required for Low dose PE Activation of Erk

Because transactivation of receptor tyrosine kinases (RTKs) was a potential mechanism of Erk activation [17], we analyzed the activation-dependent phosphorylation state of the fibroblast and general growth receptors, FGFR and EGFR. Following α_1a_-AR stimulation, phosphorylation of both RTKs exhibited temporal patterns that were recognizably similar to one another (Fig. 4C, upper panel), despite P-EGFR signal near the limit of detection. Rapid but transient phosphorylation of the RTK effector, Akt, was also observed following RTK phosphorylation but was temporally distinct from Erk activation and was lost as Erk reached maximal activity. Semi-quantitative estimation of relative band intensity (Fig. 4C, lower panel) illustrates the temporal distinctions between these activation profiles.

**Figure 5:**
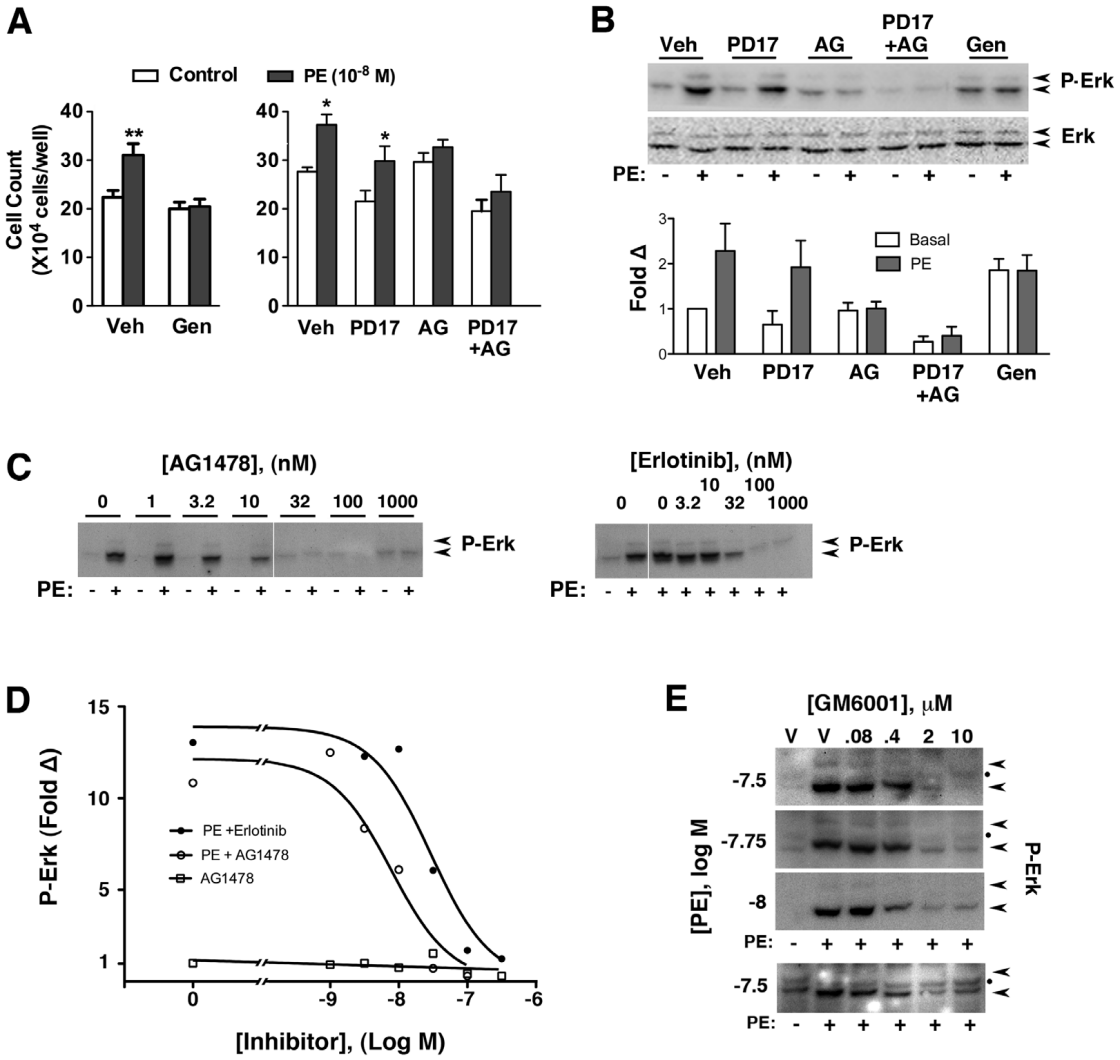
EGFR transactivation through a TMP mechanism is required for low dose α_1a_AR-induced proliferation and Erk activation. α_1a_AR expressing cells were placed into SF media prior to pretreament and addition of PE. (**A**) Prior to counting, cells were pretreated for 30 min with 0.1% DMSO (Veh), 20 µM Genestein, 1 µM PD173704 (PD17), 1 µM AG1478 (AG), 1 µM PD173704+1 µM AG1478 prior to growth without (control) or with 10^−8^ M PE. Values are the mean±SEM; (*), P<0.05 compared to vehicle or inhibitor alone (n≥3); (**), P<0.05 compared to vehicle+PE. (**B**) Representative Western analysis of Erk phosphorylation in cells pretreated for 30 min with vehicle 0.1% DMSO (Veh), 1 µM PD17, 1 µM AG, 1 µM PD +1 µM AG or 20 µM genistein (Gen) prior to stimulation without (−) or with (+) PE at 2×10^−8^ M for 10 minutes. Lower panel shows semi-quantitative analysis of P-Erk band intensity from CCD images. Values are the mean±SEM (n = 4); (*), P<0.05 compared to vehicle or inhibitor alone. (**C**) Inhibition of Erk phosphorylation by a dilution series of the EGFR inhibitors, AG1478 and Erlotinib, added 5 minutes prior to stimulation with 3×10^−8^ M PE for 5 minutes. To maintain relative intensity, panels were concordantly adjusted. (**D**) Quantitation of P-Erk band intensity at the indicated AG1478 and Erlotinib concentrations relative to levels in untreated cells. (**E**) Top 3 panels shows western analysis of Erk phosphorylation in cells pretreated for 30 min with 0.1% DMSO (Veh) or GM6001 (GM) prior to incubation for 5 minutes without (−) or with (+) PE. The bottom panel shows a similar experiment in which the GM6001 preincubation was ∼15 seconds. Panel adjusted independently to emphasis inhibition pattern. (•) Indicates an artifactual band.

Consistent with RTK involvement, the general RTK inhibitor, genistein, completely prevented the α_1a_AR induced proliferative response (Fig. 5A) as well as increased Erk phosphorylation (Fig. 5B). To identify the pathway responsible, specific inhibitors of potential RTKs were tested. While the FGFR inhibitor, PD173074, modestly decreased proliferation (Fig. 5A) and Erk phosphorylation (Fig. 5B) of both basal and stimulated cells, it did not appear to block the increase in proliferation and Erk activation induced by low doses of PE. In contrast, the EGFR inhibitor, AG1478, had little impact on either basal proliferation or Erk phosphorylation, but largely prevented both agonist-induced proliferation and Erk activation (Fig. 5A and 5B). Inclusion of both inhibitors resulted in apparently additive effects of low basal proliferation and minimal agonist-induced proliferation. Because preliminary experiments showed 100 µM concentrations of AG1478 and a second EGFR inhibitor, Erlotinib, completely blocked Erk activation even with a short 5 minute preincubation, dose response experiments were performed which demonstrated respective IC50s 8±5 nM and 30±4 nM for these inhibitors (Fig. 5C). These results are consistent with the potent EGFR inhibition previously reported [56], [57] and suggest intended target inhibition.

To demonstrate that TMP signaling was the mechanism of EGFR transactivation responsible for increased Erk activity, various concentrations of the commonly employed “general” MMP domain protease inhibitor, GM6001 (galardin), were applied to cells 30 minutes prior to PE stimulation (Fig. 5D). Across the PE concentrations that induce proliferation, GM6001 reduced receptor-activated Erk phosphorylation in a dose dependent manner. As TMP signaling is predicated on growth factor precursor proteolysis occurring outside the cell, competitive inhibition by GM6001 [58] should be almost instantaneous, as confirmed by the similar results obtained with a GM6001 preincubation of ∼15 seconds (Fig. 5D, lowest panel). These results strongly suggest TMP transactivation is essential for low dose α_1a_AR activation of Erk and increased proliferative of rat-1 cells.

### Erk Activation Requires PLCβ Production of DAG but not Increased Intracellular Calcium

**Figure 6:**
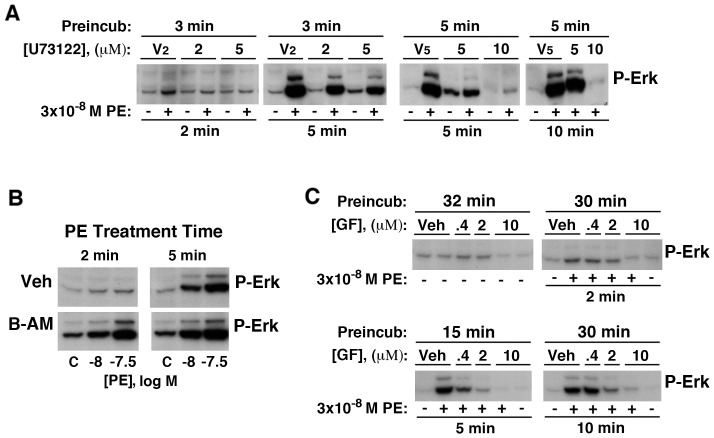
Low-dose α_1a_AR-induced proliferation requires Erk phosphorylation dependent upon Gq signaling through PLCβ and PKC. α_1a_AR expressing cells were placed in SF media prior to pretreatment and stimulation. (**A**) Western analysis of cells preincubated with DMSO at 0.1% (V2) or 0.25% (V5) or with the PLCβ inhibitor, U73122, prior to stimulation without (−) or with (+) 3×10^−8^ M PE for the indicated times. Panels adjusted independently. (**B**) Western analysis of cells preincubated 10 min with 0.2% DMSO (Veh) or with 40 µM BAPTA-AM (B-AM) prior to PE stimulation at the times and concentrations indicated. (**C**) Western analysis of cells preincubated for 15 or 30 minutes with with 0.2% DMSO (Veh) or the PKC inhibitor, GF109203X, prior to stimulation without (−) or with (+) 3×10^−8^ M PE for the indicated times.

Although the ability of Gq-coupled GPCRs to transactivate EGFR leading to Erk mediated proliferation is well established in rat-1 cells [17], [59]–[61]], the role of canonical Gq signaling in this process has not been investigated. Consistent with a requirement for Gq activation, inhibition of PLCβ with U73122, effectively blocked increased Erk phosphorylation (Fig. 6A). In these images some bands have been overexposed to allow visualization of basal Erk activity; however, these long exposures show the variable phosphorylation of the p44 Erk isoform, providing an accurate proxy for p42 phosphorylation in less exposed images. Recently, it has been reported that U73122 can also inhibit the SERCA calcium pump [62], potentially emptying ER stores and suppressing IP3R-mediated Ca^2+^ responses through a mechanism independent of Gq/PLCβ signaling. However, these authors found Gq/PLCβ/IP3R is completely inhibited by 10 µM U73122 in less than 3 minutes whereas the larger ER Ca^2+^ transients mediated by caffeine-activated ryanodine receptors was impacted more slowly (>4 min), suggesting maintenance of adequate ER Ca^2+^ levels across this period. Using a short, 3 minute, preincubation, we found the initial PE-induced increases in Erk phosphorylation were inhibited by 2 µM and blocked by 5 µM U73122 (Fig. 6A, 2 min PE). Note that basal Erk phosphorylation at that time (3 min preincubation plus 2 min incubation) was unaffected by 5 µM U73122, suggesting minimal impact on Erk signaling at the time of receptor stimulation (3 min). Erk phosphorylation following 5 minute of PE stimulation was substantially reduced by 5 µM U73122 (note p44 isoform); however, complete inhibition required a concentration of 10 µM (Fig. 6A, right panels).

To delineate the PLCβ signaling pathway responsible for transactivation we investigated dominant downstream effector pathways. In contrast to PLCβ inhibition, complete chelation of intracellular Ca^2+^ with 40 µM BAPTA-AM [63] increased basal Erk phosphorylation and did not prevent acute α_1a_AR-induced Erk phosphorylation (Fig. 6B), strongly suggesting increased cytosolic Ca^2+^ is not necessary for Erk activation as well as limiting potential problems associated with off-target SERCA inhibition by U73122 (above). On the other hand, broad spectrum inhibition of PKC isoforms with GF109203X resulted in concentration dependent reduction in Erk phosphorylation that was complete at the concentration of 10 µM whether α_1a_AR was stimulated with 3×10^−8^ M (Fig. 6C) or 10^−8^ M PE (data not shown). These results strongly suggest that Erk activation by low doses of PE requires canonical Gq signaling and is dependent on PLCβ activation presumably of novel PKC isoforms, which do not require calcium.

### α_1a_AR Induced Activation of PI3K/Akt is Separable from Erk Activation and Proliferation

**Figure 7:**
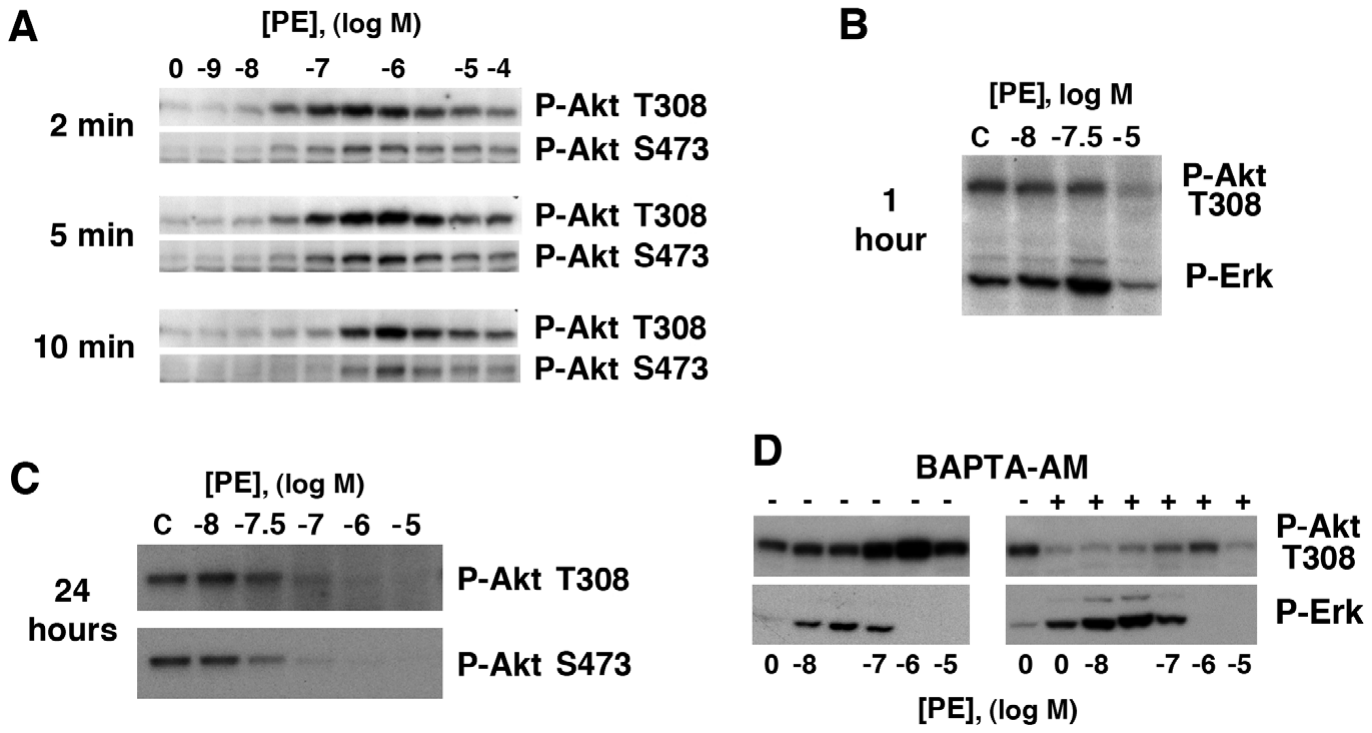
Stimulation of α_1a_AR results in strong Ca^2+^ dependent Akt activation followed by chronic Akt downregulation at high PE concentrations. α_1a_AR expressing cells were placed into SF media prior to pretreatment and stimulation with PE. Representative Western Analysis showing the PE dose dependence of (**A**) acute Akt phosphorylation from 2–10 min., (**B**) intermediate Akt and Erk phosphorylation at 1 hour and (**C**) chronic Akt dephosphorylation at 24 hours. (**D**) Treatment of cells with 0.1% DMSO vehical (−) or 20 µM BAPTA-AM (+) for 10 min. prior to 5 min stimulation with various concentration of PE demonstrate an important role for intracellular Ca^2+^ release in Akt activation that contrasts with Erk behavior.

EGFR signaling through Akt can be proliferative; however, α_1a_AR activation of Akt was temporally complex and not maximal at PE concentrations inducing proliferation. At low PE concentrations, modest, acute Akt activation returned to baseline by 15 minutes (Fig. 4C, and 7A); however, at higher PE concentrations more intense Akt activation is preserved for a longer period (Fig. 7A). These findings do not contradict an earlier report that α_1a_AR activation in rat-1 cells inhibits Akt signaling [64], as high doses of PE invariably reduced Akt phosphorylation within one hour (Fig. 7B). Indeed, chronic PE administration for 24 hours strongly inhibits Akt activity at all PE concentrations above those associated with cell proliferation (Fig. 7C). Of equal importance and in stark contrast with Erk activation, Akt phosphorylation appears strongly dependent on intracellular Ca^2+^ as BAPTA-AM inhibits basal and stimulated Akt activity (Fig. 7D). Although chronic inhibition of Akt probably plays a role in enforcing the antiproliferative phenotype, these delayed effects are beyond the scope of the current report as they are not associated with the proliferative response and probably involve distinct changes in gene expression.

## Discussion

Generally, α_1a_AR stimulation has been associated with antiproliferative [38] and [39], [65] hypertrophic phenotypes; however, this receptors ability to induce proliferative [46] and protective [66] responses demonstrates a diversity of biological functions. This variability is consistent with contradictory α_1a_AR signaling responses that are not easily distinguished from responses of the other subtypes. Consistent with phenotypic results in expression models [38], [39], pharmacologically dissection in primary foreskin fibroblasts suggests the α_1b_AR mediates proliferation rather than the equally expressed α_1a_AR. [67]. Indeed, until very recently [50], no study had reported α_1a_AR-induced EGFR transactivation, even though rat-1 cells were used in the original dissection of TMP signaling [17], [59], [60], [68] and have been a primary model of α_1a_AR signaling. Nevertheless, under some conditions the native α_1a_AR appears to enhance proliferation [46] as we observed with minimal stimulation in rat-1 fibroblasts due to a mechanism involving EGFR transactivation.

The α_1a_AR has been viewed as a stress receptor due largely to agonist-induced activation of stress pathways, including sustained increases in cytosolic Ca^2+^ [10]–[13] and activation of both p38 and Jnk [36], [37], [69], [70], all of which are associated with cell death [71] [72]. Also induced are potentially deleterious immunologic pathways including arachidonic acid release [73], NF-κB activation, IL6 secretion [74] and TNFα secretion [75] due to TACE transactivation [76] all concordant with α_1a_AR function during wound healing responses [46], [75], [77]. More broadly, Gq/PLCβ activation appears to have played a conserved role in wounding as far back as C. elegans [78].

As a stress activated kinase, with an established role in blocking the cell cycle at both the G1/S and G2/M transitions [79], it is unsurprising that the robust and extended p38 activation induced by α_1a_AR stimulation produces a nearly complete cell cycle blockade that can be prevented by p38 inhibition. Consistent with indirect blockade of the G1/S transition through Erk inhibition [79], p38 phosphorylation is also associated with a reciprocal decrease in Erk phosphorylation. At longer times, cell cycle blockade is probably reinforced by genetic reprogramming as has been suggested [38], [39], presumably through mechanisms related in part to activation of p38 and its effectors. Despite considerable study, the mechanism by which α_1a_ARs and other GPCRs activate p38 to produce an antiproliferative phenotype has yet to be established. Both our laboratory [52] and others [54] have observed no requirement for canonical PLCβ signaling in α_1a_AR-mediated p38 activation. Fairly recently it has become apparent that both Gq and Gβγ can induce RhoGEF activation of Rho GTPases [15], [80], [81] and that one pathway is mediated by direct RhoGEF association with G_q/11_ [82] or G_12/13_ [15] bypassing PLCβ. A number of studies have linked Rho GTPases to p38 activation, including a recent mechanistic description of p38 activation following α_1b_AR stimulation through the RhoGEF, AKAP-lbc [83]. Ironically, these investigators used α_1b_ARs expressed in HEK293 cells, where the α_1b_AR has an uncharacteristic antiproliferative, hypertrophic phenotype [84], perhaps because transactivation is poorly coupled in these cells [85]. The relevance of this pathway and other Rho/Gef signaling to p38 activation by the α_1a_AR and other GPCRs requires further study.

Given the usual antiproliferative and hypertrophic phenotypes, the ability of the α_1a_AR to induce proliferation of rat-1 cells through EGFR transactivation provides important support for *in vivo* evidence of this phenotype. Extensive studies in rat-1 cells have shown most Gq-coupled receptors [ET_A_, LPA, Thrombin [59], M1-acetylcholine [17], BB2-bombesin, Bradykinin [60], CASR-calcium [61]] can transactivate EGFR through a TMP mechanism involving Hb-EGF. However, until our recent report [50] and the data presented above, the α_1a_AR was a notable exception. An extensive analysis in GT1-7 neuronal cells had suggested EGFR transactivation by the α_1a_AR involved Hb-EGF release that required both PKC and Src activities [86]; however, these cells reportedly express more proliferation associated α_1b_AR [21]. Although SMCs lose α_1a_ARs during isolation [30], an elegant series of *in vivo* and vessel studies by the Faber laboratory suggest the α_1a_AR can increase proliferation of both SMCs and fibroblasts during vessel injury [46], [77], [87]. Despite the predominance of the α_1d_AR in the SMCs of conduction vessels and the presence of the proliferative α_1b_AR [31], pharmacologic dissection shows the α_1a_AR is essential for proliferation [77], [87]. Of note, the proliferative effect of the α_1a_AR on medial SMCs occurred despite the near absence of this receptor from this cell type [31], potentially suggesting cell to cell endocrine-like signaling as a result of transactivation of the fibroblast population. The α_1a_AR can also be protective as in heart [66], where activation of EGFR by Gq-coupled GPCRs including the α_1a_AR/α_1b_AR may involve EGFR transactivation and Erk signaling [88], [89]. In cardiomyocytes, Gq-coupled GPCRs activate Erk much more than PI3K/Akt [90], potentially suggesting a mechanism distinct from transactivation in which growth factor release might be expected to activate both pathways [89]. In this regard, it is notable that α_1a_ARs in rat-1 cells activate Erk at a lower concentration of agonist using fewer activated receptors, without a requirement for Ca^2+^ release.

The role of canonical Gq-coupled signaling in EGFR transactivation has received relatively little attention and had not been studied in relation to α_1a_AR signaling. More surprisingly, given the extensive study of TMP transactivation, neither has the role of canonical Gq signaling been addressed in rat-1 cells for any receptor. The results reported here suggest a requirement for PLCβ activation during EGFR transactivation by the α_1a_AR, despite minimal IP3 production [52] or calcium release [12] at the agonist concentrations that lead to Erk activation. In addition, cytosolic chelation of this minimal Ca^2+^ release does not prevent Erk phosphorylation. Combined with a requirement for PKC activity, these results suggest the essential function of PLCβ during α_1a_AR induced proliferation is DAG activation of a nonclassical PKC isoform.

Even for other Gq-coupled receptors, evidence for or against canonical signaling in transactivation is limited and disparate [19], [91], [92], however, some studies report PKC involvement upstream of EGFR activation [19], [85], [93]. Problematically, many studies have focused on the AT1R, which along with the α_1b,_ β1 and β2 adrenergic receptors, displays rapid β-arrestin mediated internalization [94] that often leads to sustained cytosolic Erk activation through intracellular βArr signaling [94], [95]. In contrast, the α_1a_AR internalizes very slowly [41], [96], [97] through a mechanism largely independent not only of the carboxy terminus [96] but also receptor activation [98], [99] and phosphorylation [100]. These characteristics suggest internalization-dependent mechanisms of Erk activation will be less important to α_1a_AR signaling perhaps favoring EGFR transactivation, particularly in rat-1 cells where signaling by focal adhesion complexes is limited [101]. More broadly, activation of PKC by DAG or phorbal esters is generally proliferative and has been implicated in transactivation [102]. Given the number of Gq-coupled receptors that can transactivate EGFR, it seems likely that DAG frequently functions as an initiator of PKC induced proliferation.

The proliferative response induced by minimal α_1a_AR stimulation occurred over a narrow and somewhat variable range of PE concentrations (10^−8^ to 3×10^−8^ M) at the lower edge of the efficacious concentrations. Consequently, 10^−8^ M PE was sometimes ineffective, leading to frequent use of the slightly higher concentration. Nevertheless, all PE concentrations producing a proliferative response were considerably below the EC_50_ of IP3 formation (PE ∼3×10^−7^ M), clearly demonstrating a tiny fraction of available receptors are responsible for the phenotype. Although distinct fractional populations of α_1a_ARs have been identified [45], [52], [103], α_1a_ARs stably expressed in rat-1 cells at ∼1.8 pmole/mg display unambiguous receptor reserve behavior including agonist binding affinities (Kd ∼10^−5^ to 3×10^−5^M) that are 30- to 100-fold above the EC_50_ for IP3 production [49], [52]. In addition, receptors at 5-fold lower density display about 5-fold higher EC_50_ values [49], implying conservation of activated receptor number. Although receptor reserve does not disprove the existence of a subpopulation of α_1a_ARs with special characteristics and high agonist affinity, it allows the possibility that fractional activation of “typical” receptors could produce the low dose response. In any case, PLCβ activated by low doses of PE drives very strong Erk activation despite almost undetectable increases in IP3 and presumably DAG at the whole cell level. This finding is of considerable significance, as it suggests Erk activation by EGFR may be the pathway most easily activated by agonist stimulation of α_1a_AR in fibroblasts.

Somewhat divergently, our recent finding that a mutant α_1a_AR (G247R) induces proliferation through constitutive EGFR transactivation suggested a mechanism that was G protein independent [50]. While G247R-α_1a_ARs may in fact bypass the requirement for PLCβ/PKC, our present data shows that very small, agonist-induced increases in IP3/DAG can induce transactivation. This readily explains how basal PLCβ activity associated with G247R-α_1a_AR was not detected, but cannot explain why prazosin did not significantly prevent G247R-α_1a_AR induced proliferation [50]. One possibility is that G247R-α_1a_AR, which constitutively activates EGFR, induces enough constitute PLCβ activity and DAG production to enable transactivation even when prazosin is bound. Alternatively, chronic transactivation by the mutant receptor may have reprogrammed gene expression using well established EGFR/Erk based mechanisms [92], resulting in a reduced requirement for PLCβ/PKC signaling. Potential divergence between acute and chronic signaling is also relevant to proliferation due to agonist-induced Erk activation; however, in the stimulated model acutely released growth factors remain in the media as evidenced by the continued elevation of FGFR phosphorylation. Delineating the importance of chronic stimulation due to acutely released growth factors from the proliferative effects of EGFR/Erk mediated gene expression seemed unlikely to be definitive and has not been pursued.

Transactivation dependent signaling by PI3K/Akt downstream of α_1_ARs [26] and other Gq-coupled GPCRs [18] appears important for contraction of smooth muscle cells. In mesenteric resistance arteries, where the α_1a_AR (or α_1L_AR) is dominant at the mRNA [43], [104] and functional [42], [105] levels, inhibition of EGFR has little impact on acute α_1_AR-mediated contraction [106] induced by Ca^2+^/Calmodulin activation of myosin light chain kinase [107], but largely prevented sustained vessel contraction induced by PI3K/Akt downstream of EGFR transactivation [106], [108]. In the current study, the role of PI3K/Akt signaling in proliferation was less clear and maximal activation of Akt by the α_1a_AR did not correlate with the low dose proliferative response. For this reason it was not a focus of the current study, nevertheless, it is noteworthy that the PI3K/Akt pathway often supports Erk signaling at low levels of EGFR activation [109] given that α_1a_AR-induced transactivation of Erk was preceded by modest, brief Akt activation.

On the other hand, robust acute Akt activation at higher PE concentrations correlated with proliferative inhibition. However, this anti-proliferative effect is more reasonably linked to chronic Akt inhibition subsequent to p38 activation. The need for Akt signaling during cellular growth suggests retention of Akt activity with low agonist plays at least a permissive role in allowing proliferation. The divergent requirement for release of intracellular Ca^2+^ for Erk and Akt activation was unexpected and clearly suggests distinct separable signaling pathways. Of potential significance to fibroblasts, α_1a_AR stimulation also resulted in transactivation of an FGFR, perhaps FGFR3, which functions primarily through PI3K/Akt signaling [110], [111]. Importantly, an inhibitor with specificity toward FGFR1/3 slightly reduced both Erk phosphorylation and cell proliferation without apparently impacting agonist-induced effects. While EGFR clearly plays an essential role in α_1a_AR agonist induced Erk activation and proliferation of rat-1 fibroblasts, the biological function of PI3K/Akt signaling requires additional study.

The combinatorial basis of stress signaling has been recognized for more than a decade in well studied models of cardiac injury [112]. Concordantly, we view the question of which pathways represent native α_1a_AR signaling as a red herring, given the array of receptors activated with the α_1a_AR during severe stress and tissue injury. In those situations where isolated α_1a_AR signaling operates as part of normal tissue function [e.g. during penile vessel contraction [113]], transactivation of Akt may represent a dominant signaling process. However, during severe stress, combinatorial signaling can induce extreme responses, such as the sustained Ca^2+^ elevation and p38 activation of cardiac ischemia [114], [115] that are similar to high dose α_1a_AR response in the rat-1 model. Less extensive vessel injury will be associated with less adrenergic stimulation and lower receptor activation that may support proliferation and vessel repair [77], [87]. Indeed, recent evidence that chronic stress responses such as cardiac hypertrophy are mediated by fibroblast activation [47], suggest additional roles for α_1a_ARs in fibroblasts [48]. While the biological reason for proliferative and antiproliferative signaling through the same receptor remains to be determined, the unanticipated isolation of Erk signaling at the lowest agonist concentrations allowed unambiguous analysis of this pathway independent of concurrent signaling through unrelated pathways or other α1AR family members.
